# Isolation of antibacterial compounds from *Quercus dilatata* L. through bioassay guided fractionation

**DOI:** 10.1186/1476-0711-11-11

**Published:** 2012-05-03

**Authors:** Maryam Jamil, Ihsan ul Haq, Bushra Mirza, Mazhar Qayyum

**Affiliations:** 1 Department of Biochemistry, Quaid i Azam University, Islamabad, Pakistan; 2 Department of Pharmacy, Quaid i Azam University, Islamabad, Pakistan; 3 PMAS Arid Agriculture University, Rawalpindi, Pakistan

**Keywords:** Absorption spectrum, Antibacterial activity, Phytochemical analysis, RP-HPLC analysis, Solvent partitioning

## Abstract

**Background:**

Four medicinal plants (*Chrozophora hierosolymitana* Spreng*, Chrysanthemum leucanthemum* L., *Ephedra gerardiana* Wall. ex Stapf*,* and *Quercus dilatata* L.) used by indigenous healers to treat various infectious diseases were selected for the present study. The major objective of the present study was isolation and characterization of antimicrobial components from the crude plant extracts using bioassay guided fractionation.

**Methods:**

Seven methanolic extracts of the four plants were screened to identify any antimicrobial agents present in them. The active crude plant extract was fractionated first by solvent partitioning and then by HPLC. Characterization of the active fractions was done by using spectrophotometer.

**Results:**

All the seven methanolic extracts showed low antifungal activity, however, when these extracts were tested for antibacterial activity, significant activity was exhibited by two extracts. The extract of aerial parts of *Q. dilatata* was most active and therefore, was selected for further analysis. Initially fractionation was done by solvent-solvent partitioning and out of six partitioned fractions, ethanol fraction was selected on the basis of results of antibacterial activity and phytochemical analysis. Further, fractionation was carried out by RP- HPLC and purified active subfractions were characterized by comparing their absorption spectra with that of the known natural products isolated from the plants of Quercus genus.

**Discussion and conclusion:**

The results suggest that this is the first report of the isolated antibacterial compounds from this genus.

## Background

Development of multi-drug resistance in pathogenic microbes and parasites and non-availability of safe antifungal drugs for systemic mycoses necessitates a search for new antimicrobial substances from other sources, including plants. The present study also aims at isolation of new natural antimicrobial compounds from some selected plants through bioassay guided isolation. Bioassay-guided isolation integrates the processes of separation of compounds in a mixture, using various analytical methods, with results obtained from biological testing. The process begins with testing an extract to confirm its activity, followed by crude separation of the compounds in the matrix and testing the crude fractions. Further fractionation is carried out on the fractions that are found to be active at a certain concentration threshold, whereas the inactive fractions are set aside or discarded. The process of fractionation and biological testing is repeated until pure compound(s) are obtained. Structural identification of the pure compound then follows. This methodology precludes overlooking novel compounds that are often missed in studies that only identify those compounds with which the investigator is familiar. Several natural compounds have been isolated by using this methodology [1-3]. The present study was designed to use bioassay fractionation for isolation of antimicrobial agents from four selected plants including *Chrozophora hierosolymitanaChrysanthemum leucanthemum, Ephedra gerardiana* and *Quercus dilatata.*For initial screening, these four selected plants were tested against seven fungal and six bacterial strains covering a broad range of microbes.

*Chrozophora hierosolymitana* is commonly known as Dyer's croton. *Chrozophora* is genus of the family Euphorbiaceae. Harraz and Abdel-Aziz [4] reported the central analgesic effect of *Chrozophora sp. Chrozophora* species are used in traditional medicine for the treatment of diverse ailments [5]. For example antifungal [6] and antiyeast [7] activities have been reported from the genus *Chrozophora*. It has also been reported to serve as a remedy for intestinal pain, for conjunctivitis as well as cicatrizant [8,9].

*Chrysanthemum leucanthemum* is commonly known as ox eye daisy. *Chrysanthemum* is a genus of family Asteraceae. Wild *Chrysanthemum* is used as an antipyretic and anti-inflammatory herb and is prescribed to clear away heat, detoxicate, subdue swelling, dissolve lumps and treat respiratory infections [10]. Hou and Jin [11] reported the use of wild *Chrysanthemum* for dizziness, headaches, hypertension, fullness in the head and blood shot, swollen, and painful eyes due to hyperactivity of liver (Liver-fire).

*Ephedra gerardiana* is commonly known as Ma Huang. *Ephedra* is a genus of non flowering seed plants belonging to the family Ephedraceae. Plants of the *Ephedra* genus have traditionally been used by indigenous people for a variety of medicinal purposes, including treatment of asthma, hay fever, and common cold [12], used in decoction as diuretic, cholagogue, antiinflammatory and vulnerary. Some species of this genus have been used to cure urinary infections [13].

*Quercus dilatata* is commonly known as holly oak. *Quercus* is a plant genus of the family Fagaceae. It is used as an astringent and antidiarrhoeal [14]. The stem bark is used to clean foul sores. The seeds are used in the treatment of diarrhoea, menorrhagia and gastrointestinal hypertrophy. It has been reported that different species of *Quercus* possess antibacterial activity [15,16], antioxidant activity [17-19] and gastroprotective effect [20]. The galls of *Quercus* are used particularly for the treatment of wounds or burns associated with bacterial infections [21].

## Methods

### Plant material and extraction

In this study, four plants (*Chrozophora hierosolymitana* Spreng*, Chrysanthemum leucanthemum* L., *Ephedra gerardiana* Wall. ex Stapf and *Quercus dilatata* L.) were collected from different regions of Pakistan. These were identified following by Professor Dr. Mir Ajab Khan and voucher specimens were deposited at the Herbarium of Pakistan, Quaid i Azam University, Islamabad. The plant tissues were macerated at room temperature for two weeks with methanol and filtered. The solvent was removed by rotary evaporator under reduced pressure and low temperature. Extraction of each plant part (leaf, stem and root) was carried out separately and all necessary precautions were adopted to avoid cross contamination. Seven crude extracts of these plants (leaf, stem and root extracts of *C. hierosolymitana,* aerial parts of *C. leucanthemum*, stem and root extracts of *E. gerardiana* and aerial parts of *Q. dilatata*) were prepared. All the extracts were stored at –20°C.

### Antifungal assay

Antifungal activity against seven fungal strains (*Fusarium moniliformes, Fusarium solani, Aspergillus niger, Aspergillus fumigatus, Aspergillus flavus, Alternaria* sp. and *Mucor* sp.) was determined using agar tube dilution method as reported earlier [22]. All fungal strains were grown on 6.5% SDA (Sabouraud dextrose agar, pH 5.7) at 28°C and preserved at 4°C in refrigerator. Screw capped test tubes containing Sabouraud dextrose agar (SDA) medium (4ml) were autoclaved at 121°C for 15 minutes. The tubes were allowed to cool at 50°C and non solidified SDA was loaded with 66.6μl of plant extracts pipeted from the stock solution (24mg/ml in DMSO) to make 400μg/ml final concentration. Tubes were then allowed to solidify at room temperature in slanting position. Each slant was inoculated with equal amount of fungal culture of size 4mm diameter and incubated at 28°C for 7-10 days. The media supplemented with DMSO and Turbinafine (200μg/ml) were used as negative and positive control respectively. The fungal growth was determined by measuring linear growth (mm) and compared with negative control to get the % age inhibition by using the following formula.

(1) $$\%\;age\;of\;fungal\;inhibition=100–\frac{Fungal\;growth\;\left(mm\right)\;in\;sample}{Fungal\;growth\;\left(mm\right)\;in\;control}\;x\;100$$

### Antibacterial assay

Antibacterial assay was carried out as reported earlier [23]. All plant extracts and fractions were tested against three gram-positive bacterial strains i.e., *Bacillus subtilis* (ATCC 6633)*, Micrococcus leuteus* (ATCC 10240)*, Staphylococcus aureus* (ATCC 6538)] and three gram negative ones i.e., *Escherichia coli* (ATTCC 1522)*, Salmonella setubal* (ATCC 19196) and *Bordetella bronchiseptica* (ATCC 4617)*.*

#### Agar well diffusion method

The agar well diffusion method, was applied for the determination of inhibition zones and minimum inhibitory concentration (MIC) of plant extracts and partitioned fractions. Briefly, 0.75ml of the broth culture containing 10^8^ colony forming units (CFU) per ml of the test strain was added to 75ml of nutrient agar medium at 45°C, mixed well, and then poured into a 14cm sterile petriplate. The medium was allowed to solidify, and 8-mm wells were dug with a sterile metallic borer. Eight concentrations of extract, two solutions for positive control (Roxithromycin and Cefixime-USP, one for each) and one for negative control (DMSO) were applied to each petriplate. These plates were incubated at 37°C. After 24 hrs of incubation the diameter of the clear zones, showing no bacterial growth, around each well was measured. Triplicate plates were prepared for each extract. Mean clear zone of these plates was calculated with standard error. The minimum inhibitory concentration (MIC) was determined by agar well diffusion [24] method. Serial dilutions of each extract (1mg/ml) in DMSO were prepared to obtain a 0.1–0.9mg/ml concentration range. A 100 μL of each dilution of the extract was introduced into wells in nutrient agar plates pre inoculated with test bacterial strains. The extracts were allowed to diffuse at room temperature before incubation at 37°C for 24 h.

#### Disc diffusion method

Disc diffusion method was applied for the determination of inhibition zones and minimum inhibitory concentration (MIC) of fractions. Filter paper discs of 6 mm diameter containing only one concentration of each fraction i.e., 1mg/mL in methanol were tested against the six bacterial strains. A 20μL of the prepared concentration of each fraction was applied to a disc. The solvent was evaporated and the disc was placed on nutrient agar plates pre inoculated with test bacterial strains.

### Fraction preparation

#### Solvent partitioning

**Figure 1:**
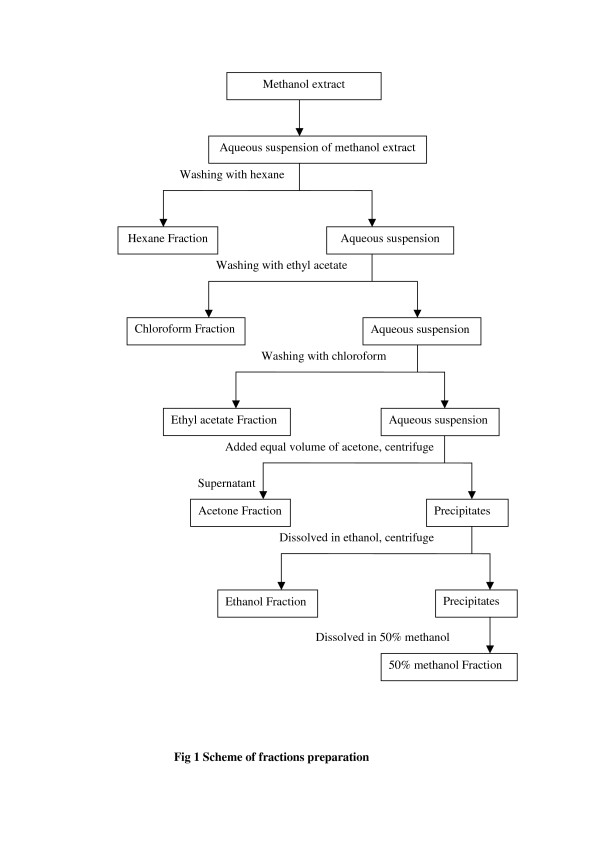
Scheme of fractions preparation.

The crude extract of the most active antibacterial plant was subjected to bio-guided fractionation by solubilisation in water and sequential partition with hexane (5 × 400mL), ethyl acetate (5 × 400mL), chloroform (3 × 400mL), acetone (5 × 400mL), ethanol (5 × 400mL) and 50% methanol (3 × 400mL) as indicated in Figure 1. Each fraction thus obtained, including the final hydromethanol fraction, was evaporated to dryness and subjected to antibacterial assay.

#### HPLC analysis of ethanol fraction

The highly active partitioned fraction (ethanol) was analyzed by HPLC system consisting of Agilent 1200 series preparative pump coupled with UV diode array detector. Optimization of mobile phase and wave length for RP- HPLC analysis of ethanol fraction of (A) *Q. dilatata* was carried out using Zorbax SB-C18 analytical column (4.6 × 150mm, 5 μm particle size, Agilent, Germany). Sample was prepared in methanol at concentration of 25 mg/mL. The sample was filtered quickly through a 0.2μm syringe filter. The injection volume was 20μL. Different mobile phases used for optimization with the isocratic flow kept constant at 1mL/min were as follows:

a) Methanol: water: acetonitrile: acetic acid (90:100:10:2).

b) Acetonitrile: water (85: 15)

c) Acetonitrile: methanol: water (75: 10: 15)

d) Methanol: acetonitrile: acetic acid (90: 10: 1)

e) Acetonitrile: methanol: ethyl acetate: toluene (59: 30: 10: 1) with 0.1% triethylamine.

f) Acetonitrile: water (60: 40)

g) Acetonitrile: methanol (70: 30)

A UV diode array detector was set at five different wave lengths i.e. 230nm, 235nm, 240nm, 245nm and 250nm. Acetonitrile: methanol (70: 30) and 230nm wavelength was selected for the preparative HPLC on the basis of results of analytical HPLC.

#### Preparative HPLC

RP- HPLC analysis of ethanol fraction of (A) *Q. dilatata* was done using LiChrospher 100 RP-C18 preparative column (25 × 250 mm, 5 μm particle size, Merck, Germany). The sample was prepared in methanol at a concentration of 50mg/mL. The injection volume was 1 mL. Acetonitrile: methanol (70: 30) and 230nm wavelength was used for the preparative HPLC. The isocratic flow was kept constant at 10mL/min.

RP- HPLC analysis of purified fractions was done using Zorbax SB-C18 analytical column (4.6 × 150 mm, 5 μm particle size, Agilent, Germany).

### Characterization of the purified active component

Thirty micrograms of the purified active component and each of four standards (Quercitrin, Gallic acid, Rutin and Ascorbic acid) were dissolved in 3mL of methanol to prepare 10μg/mL concentration. Absorption spectra of the purified active component and each of four standards (Quercitrin, Gallic acid, Rutin and Ascorbic acid) were obtained at 210-900nm by using spectrophotometer coupled with UV-diode array detector (DAD).

### Phytochemical analysis

Qualitative phytochemical analysis of partitioned fractions was carried out by using standard procedures to identify the constituents as described by Edeoga et al. [25] and Parekh and Chanda [26].

#### Alkaloids

To identify presence of alkaloids, 4mL of 1% HCl was added to the 0.25g of plant extract and then warmed and filtered. To 1ml filtrate 6 drops of Mayor’s reagents/Dragendroff reagent was added separately. Creamish precipitate/orange precipitate indicated the presence of respective alkaloids.

#### Saponins: (Frothing test)

To detect saponins, 0.5g of the plant extract was boiled in 5mL of distilled water. After cooling it was shaken vigorously to produce stable persistent froth.

#### Anthraquinones

To check presence of anthraquinones, 0.5g of the plant extract was boiled with 3mL of 1% HCl and filtered. To filtrate, 2mL of benzene was added and was shaken well. The benzene layer was removed and few drops of 10% NH_4_OH were added. Formation of pink, violet or red color indicated the presence of anthraquinones.

#### Couramins

For couramins analysis, 0.5g of moistened plant extract was taken in a test tube and covered with a filter paper moistened with 0.1N NaOH. The test tube was placed, for few minutes, in boiling water. Then the filter paper was removed and examined in UV light for yellow florescence to indicate the presence of couramins.

#### Terpenoids

*(Liebermann-Burchard reaction):* To identify presence of terpenoids, 0.5g of the plant extracxt was dissolved in 2mL of chloroform and filtered. To filtrate, equal volume of acetic acid and a drop of conc. H_2_SO_4_ were added. Blue-green ring indicated the presence of terpenoids.

#### *Flavonoids, Flavones*

To detect flavanoids and flavones, 0.5g of the extract was washed with petroleum ether. The defatted residue was dissolved in 20mL of 80% of ethanol and filtered. The filtrate was used for the following test;

a) About 3ml of the filtrate was mixed with 4ml of 1% AlCl_3_ in MeOH in a test tube. Formation of yellow colour indicated the presence of flavanols, flavones.

b) About 3ml of the filtrate was mixed with 4ml of 1% KOH. A dark yellow colour indicated the presence of flavonoids.

#### Tannins

To test tannins, 0.25g of plant extract was boiled in 10mL of distilled water and filterd. Then 1% FeCl_3_ was added to the filtrate. Browrish green or a blue-black colouration indicated the presence of tannins.

#### Phlobatannins

Deposition of a red precipitate when 0.25g of plant extract was boiled with 5mL of 1% aqueous hydrochloric acid was taken as evidence for the presence of phlobatinnins.

#### Cardiac Glycosides: (Keller – Kiliani Test)

To detect cardiac glycosides, 2mL of glacial acetic acid and few drops of 1% FeCl_3_ were added to 0.5g of plant extract. Then it was underlayed with 1mL of conc. H_2_SO_4_. Green-blue color indicated the presence of cardiac glycosides.

## Results

### Antifungal assay

All of the seven crude methanol extracts were tested against seven fungal strains Linear growth inhibition was observed. All the plant extracts showed low antifungal activity against all the seven fungal strains (Table 1). Effect of interaction of the plant extract and the fungal strain remained significant at P < 0.05.

**Table 1:** Antifungal activity of crude plant extracts against seven fungal strains

**Plant extracts**	**Percentage inhibition in linear growth**						
	*A. niger*	*A. fumigatus*	*A. flavus*	*F. moni*	*F. solani*	*Mucor sp.*	*Alternaria sp.*
(L) *C. hierosolymitana*	22.08^fghijklm^ ± 2.2	2.89^qr^ ± 2.61	32.73^cdef^± 1	23.48^efghijklm^ ± 2	18.33^ghijklmno^ ± 3.3	7.14^opqr^ ± 3.3	28.26^cdefghi^ ± 5.6
(S) *C. hierosolymitana*	9.09^nopqr^ ± 1.5	13.77^jklmnopqr^ ± 1.92	27.27^cdefghi^± 3.1	16.67^hijklmnop^ ± 7.6	6.67^opqr^ ± ±1.7	14.29^jklmnopqr^ ± ±7.4	38.04^c^ ± ±5.6
(R) *C. hierosolymitana*	34.63^cde^ ± ±1.9	13.04^klmnopqr^ ± ±3.3	27.27^cdefghi^ ± ±4.2	30.3^cdefg^± ±3.8	6.67^opqr^ ± ±1.7	7.14^opqr^ ± 4.6	25^defghijk^ ± 8.6
(A) *C. leucanthemum*	28.57^cdefgh^± 1.5	28.26^cdefgh^ ± 2.5	27.27^cdefghi^ ± 4.8	34.09^cdef^± 4.7	18.33^ghijklmno^ ± 9.3	14.29^jklmnopqr^ ± 3.6	34.78^cde^± 3.7
(S) *E. gerardiana*	28.57^cdefgh^ ± 0.75	25.36^defghij^ ± 0.7	20^ghijklmn^ ± 8.3	14.39^jklmonopq^ ± 7.2	8.33^nopqr^ ± 10	28.57^cdefgh^ ± 4.6	2.17^r^ ± 3.7
(R) *E. gerardiana*	22.08^fghijklm^ ± 1.3	23.91^efghijkl^± 2.2	53.94^b^ ± 4.4	19.69^ghijklmn^ ± 4.6	5^pqr^ ± 2.9	15.71^ijklmnop^ ± 0.8	36.96^cd^ ± ±2.2
(A) *Q. dilatata*	22.08^fghijklm^ ± 1.3	2.17^r^ ± 4.5	27.27^cdefghi^ ± 4.5	25.76^defghij^± 11	11.67^mnopqr^ ± 4.4	3.33^qr^ ± 1.3	11.96^lmnopqr^ ± 4.9
Terbinafine	100^a^ ± 0	100^a^ ± 0	100^a^ ± 0	100^a^ ± 0	100^a^ ± 0	100^a^ ± 0	100^a^ ± 0

*The data represents the mean values of three replicates. ** Letters ranging from a to r indicate respective Least Significant Difference rank orders of mean values. Values with similar letters are not significantly different from each other at p > 0.05.

### Antibacterial activity of crude plant extracts

Antibacterial activity of all the seven crude plant extracts was carried out against six bacterial strains. Our results showed that only two ((R) *C. hierosolymitana* (A) and *Q. dilatata*) out of the seven plant extracts showed antibacterial activity. Plant extract of (A) *Q. dilatata* was effective against all the bacterial strains tested at all the concentrations (Table 2). Plant extract of (R) *C. hierosolymitana* showed activity against three bacterial strains (*M. leuteus*, *B. bronchiseptica*, *S. Setubal*) at the concentrations 5-25mg/mL (Table 3). Interaction effect of the plant extract, concentration and the bacterial strain was highly significant at P < 0.05. (A) *Q. dilatata* showed the largest zone of inhibition at 25mg/mL against *B. bronchiseptica* MIC value of both the extracts which showed antibacterial activity was determined. MIC values are shown in Table 4.

**Table 2:** Activity of (A) *Q. dilatata* against six bacterial strains

	**Zone of inhibition (mm) ± S.E**					
**Conc**	***E. coli***	***S. aur***	***B. sub***	***M. leu***	***B. Bro***	***S. setubal***
**1**	9.3 ± 0.333	9.7 ± 0.33	9.7 ± 0.33	10.2 ± 0.34	11.2 ± 0.17	9.3 ± 0.33
**2**	11.1 ± 0.1	11.3 ± 0.67	11.3 ± 0.88	11.5 ± 0.53	12.8 ± 0.44	11.5 ± 0.76
**5**	12.4 ± 0.21	13.3 ± 0.67	13.5 ± 0.79	12.9^opqrst^ ± 0.95	15.7^efghij^ ± 1.03	13^opqrs^ ± 0.78
**7**	13.6 ± 0.09	15.2 ± 0.83	13.7 ± 0.33	13.6^lmnopqrs^ ± 0.83	16.8^defgh^ ± 1.2	13.6^lmnopqrs^ ± 0.8
**10**	14.7 ± 0.15	16.2 ± 0.83	14.5 ± 0.47	14.9^ghijklmno^ ± 0.95	18^cd^ ± 1.73	14.2^ijklmnopq^ ± 0.77
**15**	15.4 ± 0.15	17.2 ± 0.8	15.7 ± 0.67	15.2^efghijklm^ ± 0.83	19.8^bc^ ± 1.17	15.1^fghijklmn^ ± 0.87
**20**	16.3 ± 0.15	18.9 ± 1.1	17.2 ± 0.83	16.4^defgh^ ± 0.75	21.5^ab^ ± 1.47	16.2^defghi^ ± 0.97
**25**	17 ± 0.26	19.8 ± 1.2	18 ± 1	17^def^ ± 0.82	22.7^a^ ± 1.73	17.1^def^ ± 1.05
R	15.3 ± 0.88	27.1 ± 0.93	11.7 ± 0.33	11.5 ± 0.29	33 ± 1	11.7 ± 0.57
C	35.2 ± 0.44	38.6 ± 3.4	33.3 ± 0.33	31.8 ± 1.32	20.3 ± 0.33	32.5 ± 0.79
*E. coli:-*	*Escherichia coli*	*B. sub:-*	*Bacillus subtillus*	*B. bron:-*	*Bordetella bronchiseptica*	
*S. aur:-*	*Staphylococcus aureus*	*M. leu:-*	*Micrococcus luteus*	*S. set:-*	*Salmonella setuball*	
C	Cefixime	R	Roxithromycin	Conc	Concentrations	

The data represents the mean values of three replicates ± represents standard deviation.

Letters ranging from d to w indicate respective Least Significant Difference rank orders of mean values. Values with similar letters are not significantly different from each other at p > 0.05.

**Table 3:** Activity of (R) *C. hierosolymitana* against six bacterial strains

**Conc.**	**Zone of inhibition (mm)**					
	***M. leu***	***S. aur***	***B. sub***	***E. coli***	***B. Bro***	***S. set***
**1**	-	-	-	-	-	-
**2**	-	-	-	-	-	-
**5**	9.27 ^w^ ±0.27	-	-	-	9.5^vw^ ±0.29	11.5^stuv^ ±0.09
**7**	10.6^uvw^ ±0.32	-	-	-	10.8^tuvw^ ±0.17	13.1^nopqrs^ ±0.07
**10**	12.4^qrstu^ ±0.35	-	-	-	12.1^rstu^ ±0.09	13.9^klmnopqr^ ±0.07
**15**	12.7^pqrst^ ±0.32	-	-	-	13.17^mnopqrs^ ±0.17	14.6^hijklmnop^ ±0.06
**20**	14.1^jklmnopqr^ ±0.2	-	-	-	14.7^hijklmnop^ ±0.3	16.3^defgh^ ±0.17
**25**	15.3^efghijkl^ ±0.17	-	-	-	15.7^efghijk^ ±0.33	17.2^de^ ±0.03
R	11.5 ± 0.29	27.1 ± 0.93	11.7 ± 0.33	15.3 ± 0.88	33 ± 1	11.7 ± 0.57
C	31.8 ± 1.32	38.6 ± 3.4	33.3 ± 0.33	35.2 ± 0.44	20.3 ± 0.33	32.5 ± 0.79
*E. coli:-*	*Escherichia coli*	*B. sub:-*	*Bacillus subtillus*	*B. bron:-*	*Bordetella bronchiseptica*	
*S. aur:-*	*Staphylococcus aureus*	*M. leu:-*	*Micrococcus luteus*	*S. set:-*	*Salmonella setuball*	
C	Cefixime	R	Roxithromycin	Conc	Concentrations	

The data represents the mean values of three replicates ± represents standard deviation.

Letters ranging from d to w indicate respective Least Significant Difference rank orders of mean values. Values with similar letters are not significantly different from each other at p > 0.05.

**Table 4:** MIC of (A) *Q. dilatata* and (R) *C. hierosolymitana* against six bacterial strains

**Plant extracts**	**Minimum inhibition concentration (μg/mL)**					
	***M. leu***	***S. aur***	***B. sub***	***E. coli***	***B. bro***	***S. set***
**(A)*Q. dilatata***	400	500	300	200	300	500
**(R)*C. hierosolymitana***	4000	-	-	-	4000	3000

### Antibacterial activity and phytochemical analysis of partitioned fractions

Antibacterial activity of all the six partitioned fractions of (A) *Q. dilatata* (hexane, ethyl acetate, chloroform, acetone, ethanol and 50% methanol) was carried out against six bacterial strains. Our results showed that four of the six partition fractions i.e. ethyl acetate, acetone, ethanol and 50% methanol had antibacterial activity against all the bacterial strains tested (Table 5). However the most active partitioned fraction was ethanol as it showed largest zone of bacterial inhibition (14.3-16.7mm). Effect of interaction of two factors i.e. plant extracts and bacterial strains remained significant (P < 0.05). Ethanol fraction showed highest zone of inhibition against *S. Setubal.*

**Table 5:** Activity of partitioned fractions against six bacterial strains

**Partitioned fractions**	**Zone of inhibition (mm)**					
	***E. coli***	***S. aurs***	***B. sub***	***M. leu***	***B. bron***	***S. set***
**(A)*Q. dilatata***	9.3^l^ ±0.333	9.7^kl^ ± 0.33	9.7^kl^ ±0.33	10.2^jkl^ ±0.34	11.2^ghij^ ±0.17	9.3^l^ ±0.33
**Hexane**	-	-	-	-	-	-
**Ethyl acetate**	11.5^fghi^ ±0.28	11.2^ghij^ ±0.2	10.7^hijk^ ±0.3	9.7^kl^ ±0.3	10.7^hijk^ ±0.3	10^kl^ ±0.5
**Chloroform**	-	-	-	-	-	-
**Acetone**	12.5^ef^ ±0.3	13.9^d^ ±0.1	13.5^de^ ±0.28	14^d^ ±0.5	13.7^d^ ±0.8	12.3^f^ ±0.3
**Ethanol**	16^ab^ ±0.6	16.17^ab^ ±0.17	14.3^cd^ ±0.7	15.2^bc^ ±0.6	16.17^ab^ ±0.6	16.7^a^ ±0.3
**50% methanol**	10.5^ijk^ ±0.3	11.5^fghi^ ±0.28	11.7^fghi^ ±0.2	11.5^fghi^ ±0.3	12.5^ef^ ±0.5	12^fg^ ±0.28
R	13.5±0.3	25.4±0.3	11.2±0.4	10.8±0.4	31.5±0.8	10.5±0.3
C	32.8±0.4	35.9±0.6	31.8±0.2	29.6±0.4	18.17± 0.4	29.5±0.5
*E. coli:-*	*Escherichia coli*	*B. sub:-*	*Bacillus subtillus*		*B. bron:-*	*Bordetella bronchiseptica*
*S. aur:-*	*Staphylococcus aureus*	*M. leu:-*	*Micrococcus luteus*		*S. set:-*	*Salmonella setuball*
C	Cefixime	R	Roxithromycin		Conc	Concentrations

The data represents the mean values of three replicates ± represents standard deviation.

Letters ranging from a to k indicate respective Least Significant Difference rank orders of mean values. Values with similar letters are not significantly different from each other at p > 0.05.

Phytochemical analysis of the partitioned fractions showed the presence of different classes of the compound in different fractions. Results are given in the Table 6. Results showed that alkaloids were present in the acetone, ethanol and 50% methanol partitioned fractions at varying levels. However the ethanol partitioned fraction was particularly strongly positive for alkaloids. In addition to alkaloids it contained tannins and saponins as well.

**Table 6:** Phytochemical analysis of partitioned fractions

**Class of compounds**		**n Hexane**	**Ethyl acetate**	**Chloroform**	**Acetone**	**Ethanol**	**50% methanol**
Alkaloids	Mayer’s reagent	-	-	-	++	+++	+
	Dragendorff’s reagent	-	-	-	++	+++	+
Saponins		+	-	+	+	++	++
Anthraquinones		+	-	-	-	-	-
Coumarins		-	-	-	-	-	-
Terpenoids		Blue ring in green colour	Blue ring in green colour	-	-	-	-
Flavonoids, Flavones,		Yellow	Yellow	-	-	-	-
Hydrolysable tannins		-	++	-	++	+++	++
Phlobatannins		-	-	-	-	-	-
Cardiac Glycosides		Green blue colour	Green blue colour	-	-	-	-

+++ = Strongly positive, ++ = Moderately positive, + = Weakly positive, - = Negative, Yellow = Flavonoids, Flavones positive, Blue ring in green colour = Terpenoids positive, Red ppt. = Phlobatannins positive, Green blue colour = Cardiac Glycosides positive.

Considering most active nature and presence of maximum alkaloids, ethanol partitioned fraction was selected for further purification by using HPLC.

### HPLC fractionation

By using analytical HPLC, different mobile phases were used for optimization of conditions for fractionation with the isocratic flow kept constant at 1mL/min. Ethanol partitioned fraction was resolved in maximum peaks by using acetonitrile: methanol (70: 30) as mobile phase at 230nm. Therefore, this mobile phase was selected for the fractionation of ethanol partitioned fraction. When ethanol partitioned fraction was eluted by using acetonitrile: methanol (70: 30) as mobile phase, seven peaks were observed at 230nm. These seven fractions were collected at different retention times.

### Antibacterial activity of the fractions

Antibacterial activity of all the seven HPLC fractions (AM1, AM2, AM3, AM4, AM5, AM6 and AM7) was carried out against six bacterial strains using disc diffusion assay. Our results showed that only one of the seven fractions i.e. AM3 had antibacterial activity against all the bacterial strains tested i.e., *B. subtilis* (9.5mm)*, M. leuteus* (9mm)*, S. aureus* (9.3mm), *E. coli* (9mm)*, S. setubal* (10.1mm) and *B. bronchiseptica* (8mm). AM3 showed highest antibacterial activity against *S. setubal*. This active fraction was eluted with acetonitrile: methanol (70: 30). Three peaks were observed at 230nm. Three subfractions were collected at different retention times.

To test antibacterial activity of all the three sub fractions (AM3a, AM3b and AM3c) of fraction AM3, subfractions were dissolved in methanol. AM3a was not soluble in any solvent, while the other two subfractions AM3b and AM3c had antibacterial activity against all the bacterial strains tested (Table 7). AM3b showed highest antibacterial activity against *S. setubal*. MIC values of both the active fractions which showed antibacterial activity were determined (Table 8).

**Table 7:** Antibacterial activity of subfractions against six bacterial strains

**Sub fractions**	**Zone of inhibition**(mm)					
	***M. leu***	***S. aur***	***B. sub***	***E. coli***	***B. bro***	***S. set***
AM3a	-	-	-	-	-	-
AM3b	24 ± 0.05	28 ± 0.05	29 ± 0.03	26 ± 0.06	24 ± 0.04	30 ± 0.03
AM3c	20 ± 0.05	24 ± 0.04	23 ± 0.1	24 ± 0.07	20 ± 0.03	24 ± 0.02
C	31 ± 0.07	31 ± 0.2	25 ± 0.1	33 ± 0.06	25 ± 0.1	25 ± 0.5
R	12 ± 0.1	23 ± 0.07	9 ± 0.06	11 ± 0.2	19 ± 0.05	10 ± 0.1
*E. coli:-*	*Escherichia coli*	*B. sub:-*	*Bacillus subtillus*	*B. bron:-*	*Bordetella bronchiseptica*	
*S. aur:-*	*Staphylococcus aureus*	*M. lut:-*	*Micrococcus luteus*	*S. set:-*	*Salmonella setuball*	
C	Cefixime	R	Roxithromycine			

The data represents the mean values of three replicates ± represents standard deviation.

**Table 8:** MIC of active subfraction against six bacterial strains

**Fractions**	**Minimum inhibition concentration (μg/mL)**					
	***E. coli***	***S. aur***	***B. sub***	***M. leu***	***B. bro***	***S. set***
**AM3b**	90	100	100	90	300	70
**AM3c**	200	300	500	200	500	500
**Cefixime**	30	10	50	50	80	50
**Roxithromycine**	800	100	600	600	70	600

**Figure 2:**
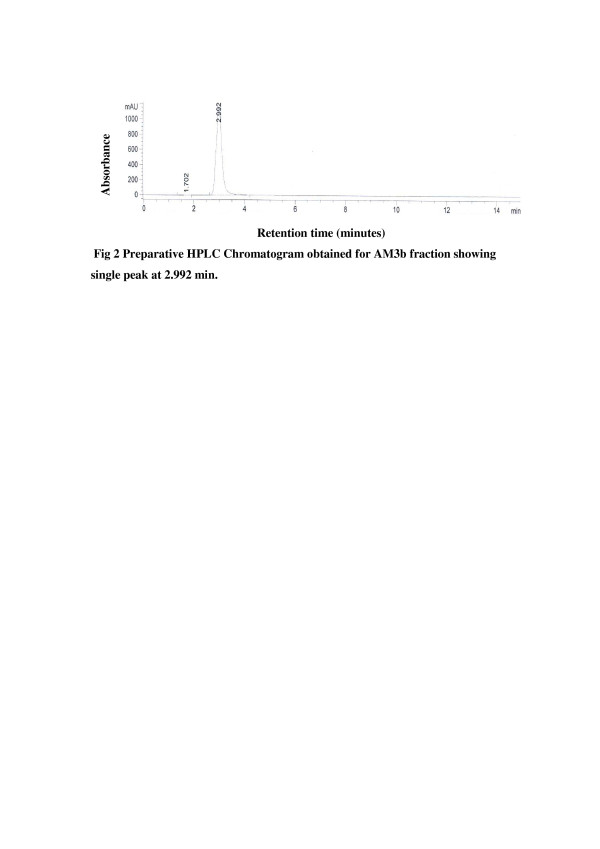
Preparative HPLC Chromatogram obtained for AM3b fraction showing single peak at 2.992 min.

**Figure 3:**
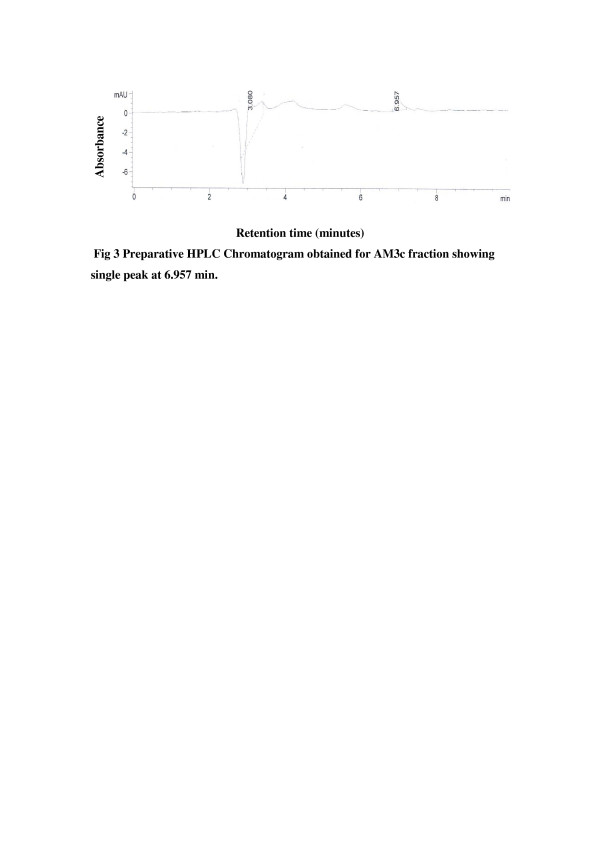
Preparative HPLC Chromatogram obtained for AM3c fraction showing single peak at 6.957 min.

Purity of the two active subfractions AM3b and AM3c was further checked by analytical RP- HPLC analysis and both the fractions showed single peak chromatogram (Figure 2, Figure 3).

### Characterization of purified active component

**Figure 4:**
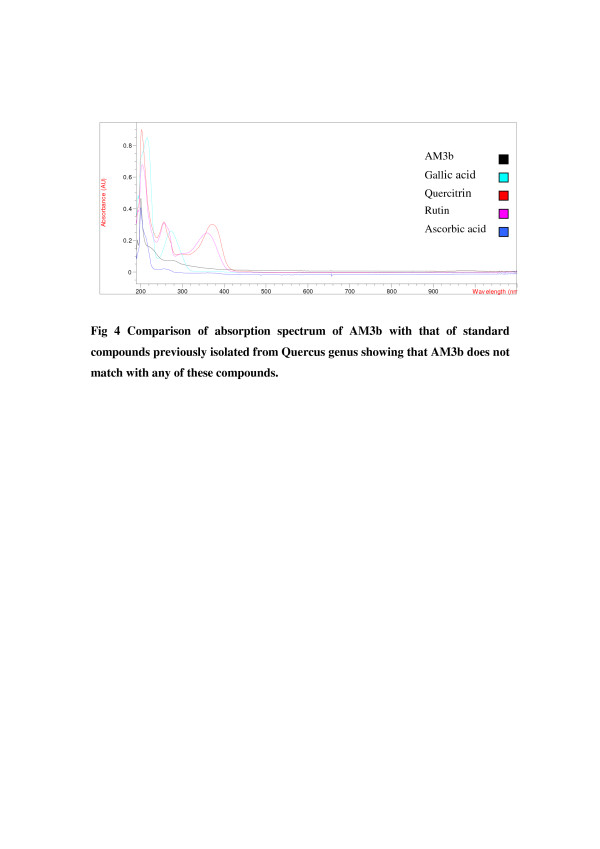
Comparison of absorption spectrum of AM3b with that of standard compounds previously isolated from Quercus genus showing that AM3b does not match with any of these compounds.

**Figure 5:**
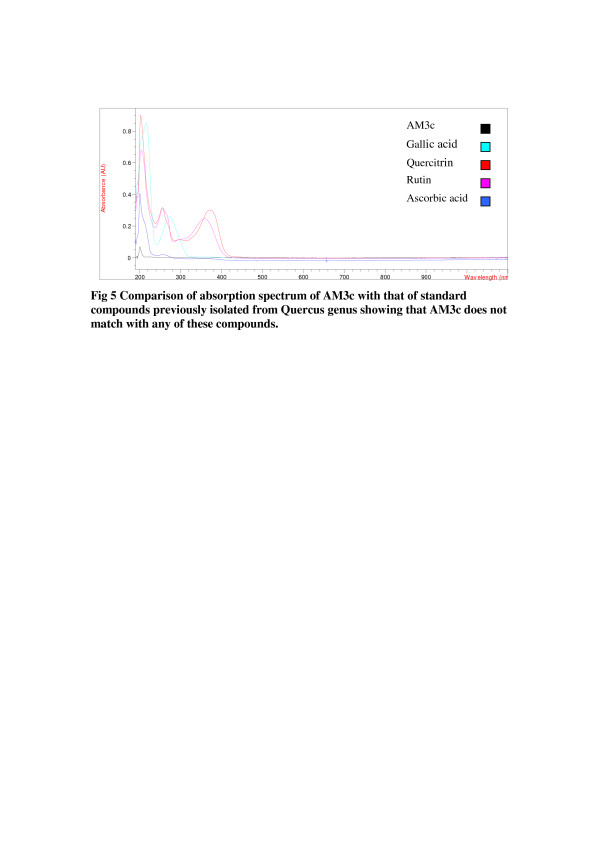
Comparison of absorption spectrum of AM3c with that of standard compounds previously isolated from Quercus genus showing that AM3c does not match with any of these compounds.

Purified active subfractions were characterized on the basis of absorption spectrum. The results showed that these two fractions contain independent constituents. Absorption spectra of active subfractions were compared with the absorption spectrum of known compounds previously isolated from the same genus (Figure 4 and Figure 5).

## Discussion

Initial screening of plants for possible active natural products typically begins by using crude aqueous or alcohol extraction and can be followed by various organic extraction methods. However, nearly all of the identified antimicrobial components from plants are aromatic or saturated organic compounds, they are often obtained through initial ethanol or methanol extraction [27]. Therefore, in the present study, for the preparation of all the seven crude extracts, methanol was used as extraction solvent. For initial screening, these seven crude methanol extracts were tested against seven fungal and six bacterial strains covering a broad range of microbes. All the extracts showed low level of antifungal activity. However, antibacterial assay of the crude methanolic extracts showed that only two extracts; aerial parts of *Q. dilatata* and roots of *C. hierosolymitana* had antibacterial activity. The extract of aerial parts of *Q. dilatata* was found highly effective against all the bacterial strains tested i.e., *E. coli, B. subtilusS. aureus, M. luteus, B. bronchiseptica,* and *S. setubal* while root extract of *C. hierosolymitana* showed antibacterial activity against only three bacterial strains i.e., *M. luteus, B. bronchiseptica,* and *S. setubal.* Previously, there have been some reports of the antibacterial and antifungal activities of different species of *Quercus*[28-31]. However, ours is the first report of antimicrobial activity of *Q. dilatata*.

Based on the results of initial screening the methanol extract of *Q. dilatata* was subjected to bio-guided fractionation by solubilisation in water and sequential partition with different solvents with increasing polarity; hexane, chloroform, ethyl acetate, acetone, ethanol and 50% methanol yielding six fractions. All the fractions were subjected to antibacterial assay. Four of the six partitioned fractions i.e. ethyl acetate, acetone, ethanol and 50% methanol showed antibacterial activity against all the bacterial strains tested whereas the hexane and chloroform extracts were inactive. Highest antibacterial activity was shown by ethanol fraction. Phytochemical analysis of partitioned fractions showed the presence of alkaloids in the three fractions in the order of, ethanol partitioned fraction > acetone partitioned fraction > 50% methanol partitioned fraction. Alkaloids are commonly known antibacterial agents [32]. Therefore, our results suggested that the ethanol partitioned fraction contained maximum alkaloids which could be responsible for the highest antibacterial activity exhibited by this fraction. The polarity of a solvent plays an important role on composition of an extract and hence on its potential antibacterial activity. Similar results were reported by Berahou et al. [33] that only ethyl acetate, butanol and aqueous phases of methanol extract of *Quercus ilex* bark showed antibacterial activity against all the bacterial strains whereas the hexane and dichloromethane phases were almost inactive.

HPLC analysis of the most active partitioned fraction i.e., ethanol was done by isocratic RP-HPLC system consisting of Agilent 1200 series preparative pump coupled with UV diode array detector. HPLC has extensively been used for isolation and identification of active natural components components [34-36]. In this study ethanol partition fraction was eluted with acetonitrile: methanol (70: 30) and seven peaks were observed at 230nm. These seven fractions were collected at different retention times. Only one of the seven fractions i.e. AM3 had antibacterial activity against all the bacterial strains tested. AM3 showed highest antibacterial activity against *S. setubal*. RP- HPLC analysis of active fraction AM3 was done. When fraction AM3 was subjected to RP-HPLC analysis, three subfractions were collected at different retention times. Two of the three subfractions AM3b and AM3c showed antibacterial activity against all the bacterial strains tested.

Purified active subfractions were charaterized by comparing their absorption spectra with that of standard natural products. In the present study, four standard natural compounds used were ascorbic acid, quercitrin, gallic acid and rutin. All of them have been reprted previously from genus *Quercus* i.e., ascorbic acid [37], quercitrin [38], gallic acid [29] and rutin [39]. The absorption spectra of the active fractions were different from that of the standard compounds previously isolated from the *Quercus* genus suggesting that these are different compounds. However, structure elucidation is required to confirm their novelty. Antibacterial [15,16] activity of genus *Quercus* was previously reported, yet antibacterial activity of *Q. dilatata* had never been checked before. Thus, this is the first report of identification and successful isolation of antibacterial compounds from this species. These compounds are effective against *B. subtilis, M. leuteus, S. aureus E. coli, S. setubal* and *B. bronchiseptica*. These bacterial strains are involved in causing many diseases like boils, cellulitis folliculitis, furuncles, carbuncles, pneumonia, meningitis, urinary tract infections etc. Furthermore, these compounds appear to be different from already known constituents of this genus and actually could be newly identified compoundsand hence can be potentially used against the above mentioned diseases.

## Conclusion

This study provides new scientific information about *C. hierosolymitana* and *Q. dilatata* based on its biological potential and phytochemical analysis that has never been reported earlier. (A) *Q. dilatata* showed promising antibacterial activities. After solvent partitioning of (A) *Q. dilatata,* ethanol fraction was selected on the basis of results of antibacterial assay and phytochemical analysis for further fractionation through RP-HPLC analysis. The compounds purified by RP-HPLC were characterized by comparing their absorption spectra with that of the known compounds isolated from the genus *Quercus* and were found to be different. In order to reveal the structure of isolated compounds, NMR and crystallographic analysis are under process.

## Competing interests

The author(s) declare that they have no competing interest.

## Authors' contributions

All the experimental design was made by first author and in experimentation all the authors contribute equally. All authors read and approved the final manuscript.
